# Prognostic implications of atrial vs. ventricular functional tricuspid regurgitation

**DOI:** 10.1093/ehjci/jead016

**Published:** 2023-02-10

**Authors:** Xavier Galloo, Marlieke F Dietz, Federico Fortuni, Edgard A Prihadi, Bernard Cosyns, Victoria Delgado, Jeroen J Bax, Nina Ajmone Marsan

**Affiliations:** Department of Cardiology, Leiden University Medical Centre, Albinusdreef 2, 2330RC Leiden, The Netherlands; Department of Cardiology, Vrije Universiteit Brussel (VUB), Universitair Ziekenhuis Brussel (UZ Brussel), Laarbeeklaan 101, 1090 Brussels, Belgium; Department of Cardiology, Leiden University Medical Centre, Albinusdreef 2, 2330RC Leiden, The Netherlands; Department of Cardiology, Leiden University Medical Centre, Albinusdreef 2, 2330RC Leiden, The Netherlands; Department of Cardiology, San Giovanni Battista Hospital, Via Massimo Arcamone, 06034 Foligno PG, Italy; Department of Cardiology, Leiden University Medical Centre, Albinusdreef 2, 2330RC Leiden, The Netherlands; Hartcentrum, Ziekenhuis Netwerk Antwerpen (ZNA) Middelheim, Lindendreef 1, 2020 Antwerp, Belgium; Department of Cardiology, Vrije Universiteit Brussel (VUB), Universitair Ziekenhuis Brussel (UZ Brussel), Laarbeeklaan 101, 1090 Brussels, Belgium; Department of Cardiology, Leiden University Medical Centre, Albinusdreef 2, 2330RC Leiden, The Netherlands; Heart Institute, Hospital University Germans Trias i Pujol, Carretera de Canyet, s/n, 08916 Badalona, Spain; Department of Cardiology, Leiden University Medical Centre, Albinusdreef 2, 2330RC Leiden, The Netherlands; Heart Centre, University of Turku and Turku University Hospital, Kiinamyllynkatu 4-8, 20521 Turku, Finland; Department of Cardiology, Leiden University Medical Centre, Albinusdreef 2, 2330RC Leiden, The Netherlands

**Keywords:** survival, tricuspid valve, functional tricuspid regurgitation, atrial functional tricuspid regurgitation, ventricular functional tricuspid regurgitation

## Abstract

**Aims:**

Atrial functional tricuspid regurgitation (AFTR) has shown distinctive pathophysiological and anatomical differences compared with ventricular functional tricuspid regurgitation (VFTR) with potential implications for interventions. However, little is known about the difference in long-term prognosis between these two FTR-aetiologies, which was investigated in the current study.

**Methods and results:**

Patients with severe FTR were divided into two aetiologies, based on echocardiography: AFTR and VFTR. VFTR was further subdivided into (i) left-sided cardiac disease; (ii) pulmonary hypertension; and (iii) right ventricular dysfunction. Long-term mortality rates were compared and independent associates of all-cause mortality were investigated.

A total of 1037 patients with severe FTR were included, of which 129 patients (23%) were classified as AFTR and compared with 425 patients (78%) classified as VFTR and in sinus rhythm. Of the 425 VFTR patients, 340 patients (61%) had left-sided cardiac disease, 37 patients (7%) had pulmonary hypertension, and 48 patients (9%) had right ventricular dysfunction. Cumulative 10-year survival rates were significantly better for patients with AFTR (78%) compared with VFTR (46%, log-rank *P* < 0.001). On multivariable Cox regression analysis, VFTR as well as all VFTR subtypes were independently associated with worse overall survival compared with AFTR (HR: 2.292, *P* < 0.001 for VFTR).

**Conclusion:**

Patients with AFTR had significantly better survival as compared with patients with VFTR, as well as all VFTR subtypes, independently of other clinical and echocardiographic characteristics.

## Graphical Abstract

**Graphical Abstract:**
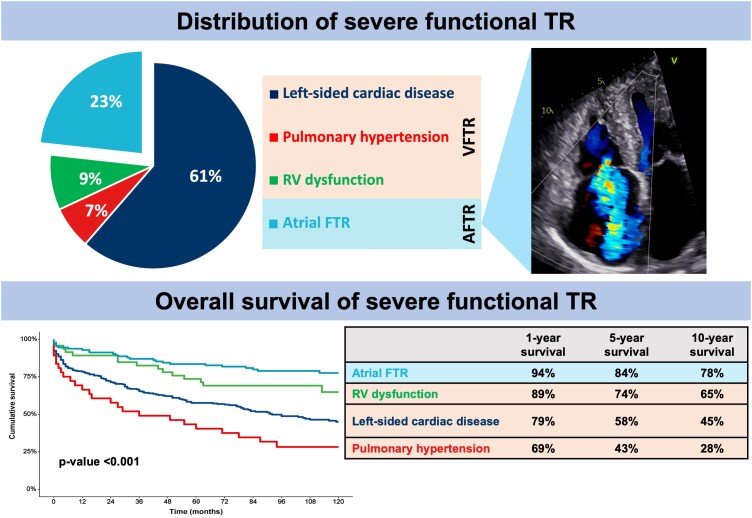
Distribution of severe FTR and overall survival according to the FTR-aetiology. AFTR, atrial functional tricuspid regurgitation; FTR, functional tricuspid regurgitation; RV, right ventricle; TR, tricuspid regurgitation; VFTR, ventricular functional tricuspid regurgitation.

## Introduction

Tricuspid regurgitation (TR) is common in the overall population, with a prevalence of 4% of clinically relevant TR in patients aged over 75 years.^1^ The prognostic implications of significant TR have been reported in several patient cohorts and if left untreated, severe TR is associated with poor survival, regardless of left ventricular (LV) function, right ventricular (RV) function, and pulmonary hypertension.^1–4^

Only 8–10% of patients diagnosed with TR present with anatomical abnormalities of the tricuspid valve (TV) apparatus (primary or organic TR), while the majority of patients show structurally normal TV leaflets, being TR caused by annular dilatation and leaflet tethering (secondary or functional TR; FTR). According to the latest literature,^5–8^ two distinct aetiologies of FTR can be identified: (i) RV remodelling, including chamber dilatation and/or dysfunction, accompanied by significant TV annular dilatation and leaflet tethering (ventricular FTR; VFTR); (ii) prominent right atrial (RA) dilatation without significant RV remodelling, leading to TV leaflet malcoaptation mainly by annular dilatation (atrial FTR; AFTR). The relatively novel entity of AFTR, formerly identified as idiopathic or isolated TR, has recently been the focus of several studies exploring the anatomical and pathophysiological differences compared with VFTR, and distinct RA and RV echocardiographic differences have now been identified.^9–12^ However, the prognostic implications of AFTR have only partially been evaluated, with only few studies published with either small cohort of AFTR patients^13^ or focusing on the comparison between organic and functional TR, or between patients receiving transcatheter TV repair vs. conservatively treated patients.^14,15^ Therefore, the aim of the current study was to evaluate in a large cohort of patients, diagnosed with severe FTR and conservatively treated, whether difference in long-term survival can be observed among different FTR-aetiologies (AFTR vs. VFTR), defined according to currently recommended clinical and echocardiography criteria and adjusted for other possible confounding factors.

## Methods

### Study population

**Figure 1:**
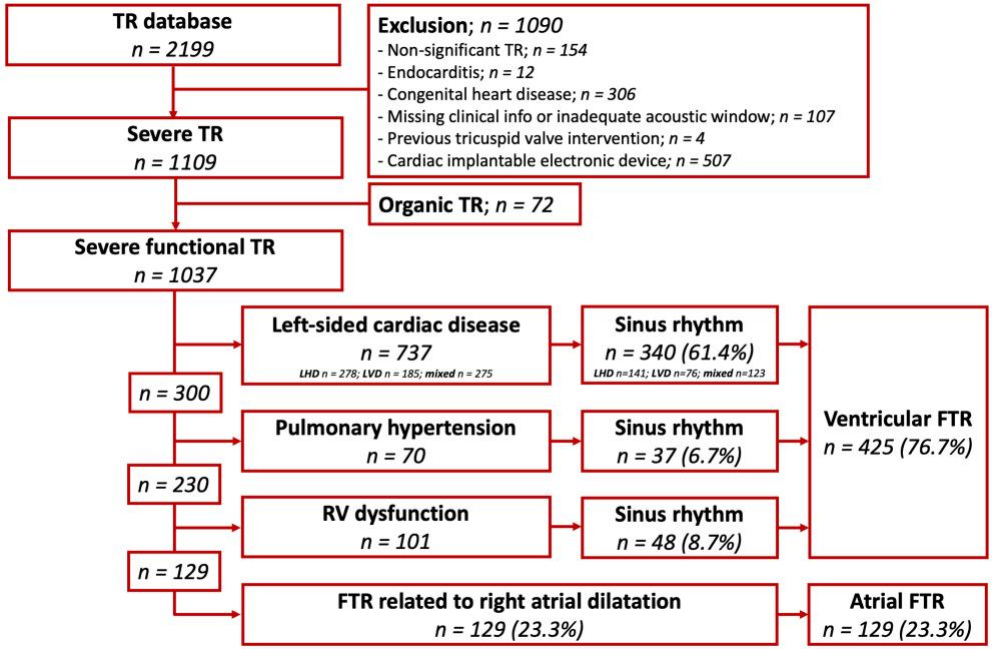
Flowchart for study population selection. FTR, functional tricuspid regurgitation; LHD, left-sided heart disease; LVD, left-sided valvular disease; TR, tricuspid regurgitation.

Patients diagnosed with severe TR between January 2000 and September 2016 were identified from the departmental adult echocardiography database of the Leiden University Medical Centre (Leiden, The Netherlands). Patients with active endocarditis, known congenital heart disease, missing relevant clinical data, inadequate echocardiographic acoustic window, prior TV intervention, or a cardiac implantable electronic device were excluded from the current analysis. In addition, patients with organic TR were also excluded (*Figure 1*).

Demographic and clinical data were retrospectively collected from the departmental Cardiology Information System (EPD-Vision, Leiden University Medical Centre, Leiden, The Netherlands). The institutional review board of the Leiden University Medical Centre approved the observational design and retrospective analysis of clinically acquired data, and waived the need for patient written informed consent. The study was conducted in accordance with the principles of the Helsinki Declaration.

### Clinical and echocardiographic variables

Baseline demographic, clinical, laboratory, and echocardiographic variables were evaluated at the time of first diagnosis of severe TR on transthoracic echocardiography. Demographic characteristics included age, sex, and body mass index. Clinical characteristics included cardiovascular risk factors, relevant medical history and comorbidities, functional status (New York Heart Association functional class), and medications.

Transthoracic echocardiograms were performed at rest using available equipment (Vivid 7, E9 and E95 systems, GE-Vingmed, Horten, Norway) and images were digitally stored for offline analysis (EchoPAC version 113.0.3, 202, 203, and 204; GE-Vingmed, Horten, Norway). All echocardiograms were prospectively re-analysed and all variables described in the current study were measured from the original digitalized echocardiography. M-mode, two-dimensional and colour, continuous- and pulsed-wave Doppler data were acquired from the parasternal, apical, and subcostal views, according to current guidelines.^16–18^ From the apical four- and two-chamber views, LV volumes were measured and indexed for body surface area, and LV ejection fraction was quantified using the biplane Simpson’s method.^16^ Left atrial maximum volume was measured at end-systole using the biplane method on the apical four- and two-chamber views and indexed for body surface area.^16^ RV measurements were performed on a RV-focused apical view. RV end-systolic and end-diastolic areas were traced and indexed for body surface area, and RV fractional area change was derived. Additionally, RV function was evaluated by tricuspid annular plane systolic excursion, measured on M-mode recordings of the lateral tricuspid annulus. RA size was measured at end-systole on an apical four-chamber view (RA volume was calculated using the single-plane disk summation method) and indexed for body surface area. Furthermore, integrative assessment of the TR severity was performed through a multiparametric approach including qualitative, semiquantitative, and quantitative parameters measured on colour, continuous- and pulsed-wave Doppler data and were graded according to current EACVI/ASE guidelines.^17,18^ Systolic pulmonary artery pressure was estimated from the TR jet peak velocity applying the Bernoulli equation and adding RA pressure. RA pressure was estimated based on the inferior vena cava diameter and its collapsibility during breathing.^16^

### Classification of FTR-aetiology

FTR was classified, using a stepwise approach, into the following four aetiologies based on clinical and echocardiographic characteristics as recently proposed^5–8,19^: (i) left-sided cardiac disease; (ii) pulmonary hypertension; (iii) RV dysfunction; and (iv) AFTR. First, patients were categorized as having *left-sided cardiac disease* when having either left-sided heart disease (i.e. LV ejection fraction <50% due to ischaemic heart disease or cardiomyopathy), or left-sided valvular disease (i.e. at least moderate mitral regurgitation or moderate aortic stenosis), or mixed left-sided disease (i.e. the combination of left heart disease and left-sided valvular disease). Next, patients with an estimated systolic pulmonary artery pressure ≥50 mmHg on Doppler echocardiography were classified as having *pulmonary hypertension*. Thereafter, patients were categorized as having *RV dysfunction* in the presence of a moderate or severe RV dysfunction, defined as a RV fractional area change <30% and/or tricuspid annular plane systolic excursion <15 mm. Finally, FTR with no other cause of annular dilatation and impaired leaflet coaptation than RA dilatation [related to atrial fibrillation (AF) or heart failure with preserved ejection fraction] were classified as *AFTR*. Aetiologies 1–3 were grouped as *VFTR* whereas AFTR remained an independent group. Importantly, to avoid potential confusion in the true aetiology of VFTR in the presence of AF, only patients with VFTR in sinus rhythm were included in the final analysis (*Figure 1*).

### Follow-up and outcome definition

The primary endpoint of the study was all-cause mortality. Outcomes were analysed from the time of diagnosis of severe TR until death or last follow-up in May 2022. Additionally, patients were censored at the moment of TV intervention. Survival data were ascertained from the departmental Cardiology Information System and the Social Security Death Index and were complete for all patients included in the study.

### Statistical analysis

Continuous variables with a Gaussian distribution are presented as mean ± standard deviation and continuous variables without a Gaussian distribution are presented as median and interquartile range. Categorical variables are presented as frequencies and percentages.

Differences between VFTR and AFTR were compared using the independent-samples Student’s *t*-test for continuous variables with normal distribution, or the Mann–Whitney *U* test for non-normally distributed continuous variables, whereas categorical variables were compared using the Pearson chi-square test or Fisher exact test, as appropriate. Differences among the four FTR-aetiologies were analysed using the one-way analysis of variance for continuous variables with normal distribution, the Kruskal–Wallis test for non-normally distributed continuous variables, and the Pearson chi-squared test for categorical variables. Multiple comparisons for continuous variables were tested with the Bonferroni correction.

The Kaplan–Meier survival analysis was used to estimate the 10-year survival rate, and differences between groups were analysed using the log-rank test. To investigate the association between clinical and echocardiographic factors with all-cause mortality, uni- and multivariable Cox proportional hazards regression analyses were performed. Variables that were significant on the univariable analysis (*P* < 0.05) were included in the multivariable regression analysis. The variables, which were used to classify the FTR-aetiologies, were not included in the model to avoid multicollinearity. Correlation factor analysis was used to determine if any pairs of variables were correlated and no collinearity (correlation coefficient >0.60) was detected for the continuous variables that met the entry criteria for multivariable regression analysis. The hazard ratios and 95% confidence intervals were calculated.

All *P*-values were two-sided, and *P*-values < 0.05 were considered statistically significant. All data were analysed using SPSS for Windows, version 25.0 (IBM Corp, Armonk, NY, USA) and R version 4.4.1717 (R Foundation for Statistical Computing, Vienna, Austria).

## Results

### Distribution of FTR-aetiologies

A total of 1039 patients, diagnosed with severe FTR, were included in the current analysis, of which 12.5% were classified as AFTR and compared with 425 patients classified with VFTR in sinus rhythm (483 patients excluded with AF) and distinguished in left-sided cardiac disease (*n* = 340, 61.4%), pulmonary hypertension (*n* = 37, 6.7%), and RV dysfunction (*n* = 48, 8.7%) (*Figure 1*).

### Clinical characteristics

The clinical characteristics of the overall population, as well as stratified according to VFTR and AFTR, are presented in *Table 1*. Mean age was 66 ± 14 years, with 231 males (42%). No significant differences between VFTR and AFTR were detected for age or sex.

**Table 1:** Baseline characteristics of the overall population and according to atrial and ventricular FTR

Clinical characteristics	Overall population *n* = 554	Ventricular FTR *n* = 425	Atrial FTR *n* = 129	*P*-value
Demographic characteristics				
ȃAge, years	66 ± 14	66 ± 15	67 ± 14	0.427
ȃMale sex, *n*(%)	231 (42)	186 (44)	45 (35)	0.083
ȃBody mass index, kg/m^2^	25.4 ± 4.5	25.4 ± 4.6	25.6 ± 4.0	0.640
Medical history				
ȃArterial hypertension, *n*(%)	365 (69)	284 (70)	81 (63)	0.155
ȃDiabetes mellitus, *n*(%)	96 (18)	83 (21)	13 (10)	**0**.**008**
ȃDyslipidaemia, *n*(%)	214 (43)	180 (47)	34 (30)	**0**.**001**
ȃSmoking, *n*(%)	165 (33)	135 (35)	30 (26)	0.072
ȃCoronary artery disease, *n*(%)	197 (36)	170 (41)	27 (21)	**<0**.**001**
ȃAtrial fibrillation, *n*(%)	58 (11)	0 (0)	58 (45)	**<0**.**001**
ȃChronic kidney disease, *n*(%)	89 (17)	71 (17)	18 (14)	0.495
ȃChronic haemodialysis, *n*(%)	13 (2)	11 (3)	2 (2)	0.742
ȃCOPD, *n*(%)	82 (15)	60 (15)	22 (17)	0.485
ȃNYHA III/IV, *n*(%)	229 (45)	205 (52)	24 (20)	**<0**.**001**
ȃPrevious cardiac surgery, *n*(%)	191 (35)	157 (37)	34 (24)	**0**.**027**
Laboratory values				
ȃHaemoglobin, g/dL	12.3 ± 2.2	12.1 ± 2.3	12.8 ± 1.9	**0**.**001**
ȃeGFR-MDRD, mL/min/1.73 m^2^	70.9 ± 31.6	69.0 ± 32.4	77.2 ± 27.9	**0**.**008**
Medication				
ȃBeta-blocker, *n*(%)	253 (51)	200 (53)	53 (46)	0.203
ȃRAAS-inh, *n*(%)	246 (50)	198 (53)	48 (42)	0.055
ȃMRA, *n*(%)	84 (17)	68 (18)	16 (14)	0.325
ȃLoop diuretic, *n*(%)	253 (48)	212 (52)	41 (33)	**<0**.**001**

Values are mean ± SD, median(IQR), or *n*(%). *P*-values < 0.05 were considered statistically significant and are shown in bold.

COPD, chronic obstructive pulmonary disease; eGFR-MDRD, estimated glomerular filtration rate—modification of diet in renal disease; FTR, functional tricuspid regurgitation; MRA, mineralocorticoid receptor antagonist; NYHA, New York Heart Association functional class; RAAS-inh, Renin-angiotensin-aldosterone system inhibitors.

Patients with VFTR had more comorbidities compared with patients with AFTR, with more cardiovascular risk factors (more diabetes mellitus, dyslipidaemia, and trend towards more smoking, leading to more coronary artery disease), worse estimated glomerular filtration rate, and lower haemoglobin levels. Patients with VFTR were also more often symptomatic (higher percentage had New York Heart Association functional class III/IV) and were treated more frequently with loop diuretics. Furthermore, a total of 58 patients (45%) from the AFTR cohort had a history of AF, while, as per selection criteria, no patients with VFTR had AF. The clinical characteristics according to the FTR-aetiology subtypes are shown in Supplementary material online, *Table S1*, which confirmed limited differences between the VFTR subtypes, and namely significant differences between AFTR and left-sided cardiac disease VFTR.

### Echocardiographic characteristics

The echocardiographic characteristics of the overall population, as well as stratified according to VFTR and AFTR, are presented in *Table 2*. In the overall population, mean LV ejection fraction was 48 ± 16% and most patients showed mildly dilated RV, normal RV function, and moderate-to-severely dilated RA.

**Table 2:** Echocardiographic characteristics of the overall population and according to atrial and ventricular FTR

Echocardiographic characteristics	Overall population *n* = 554	Ventricular FTR *n* = 425	Atrial FTR *n* = 129	*P*-value
Left-sided cardiac variables				
ȃLV end-diastolic volume-indexed, mL/m^2^	56.6 (42.9–81.3)	60.0 (44.0–88.5)	52.3 (38.8–64.0)	**<0**.**001**
ȃLV end-systolic volume-indexed, mL/m^2^	27.8 (18.3–45.1)	32.2 (20.9–55.6)	19.7 (15.4–26.2)	**<0**.**001**
ȃLV ejection fraction, %	48.1 ± 16.0	44.2 ± 16.0	60.6 ± 6.8	**<0**.**001**
ȃLeft-sided valvular disease, *n*(%)	199 (36)	199 (47)	0 (0)	**<0**.**001**
ȃLeft atrial volume max-indexed, mL/m^2^	39.7 (27.4–54.1)	40.8 (28.6–57.0)	36.0 (23.7–46.7)	**0**.**002**
Right-sided cardiac variables				
ȃRV basal diameter, mm	44.7 ± 8.6	44.8 ± 8.6	44.1 ± 8.5	0.433
ȃRV mid diameter, mm	34.6 ± 8.9	35.2 ± 9.0	32.9 ± 8.4	**0**.**011**
ȃRV base-to-apex length, mm	72.0 ± 11.6	72.9 ± 12.0	69.3 ± 10.1	**0**.**001**
ȃRV end-diastolic area-indexed, cm^2^/m^2^	12.9 ± 4.1	13.3 ± 4.2	11.6 ± 3.4	**<0**.**001**
ȃRV fractional area change, %	35.9 ± 13.5	33.0 ± 13.2	45.6 ± 8.9	**<0**.**001**
ȃTAPSE, mm	16.6 ± 5.5	15.5 ± 5.2	20.5 ± 4.6	**<0**.**001**
ȃSystolic pulmonary artery pressure, mmHg	43.0 ± 16.7	45.9 ± 17.6	33.7 ± 8.6	**<0**.**001**
ȃRA area max-indexed, cm^2^/m^2^	12.6 ± 4.0	12.2 ± 3.8	14.0 ± 4.5	**<0**.**001**
ȃRA major axis, cm	5.7 ± 1.0	5.6 ± 0.9	6.0 ± 1.1	**<0**.**001**
ȃRA minor axis, cm	4.7 ± 1.0	4.7 ± 0.9	5.0 ± 1.1	**0**.**002**
ȃRA volume max-indexed, mL/m^2^	36.8 (27.1–52.4)	35.8 (25.2–50.9)	40.8 (32.2–57.5)	**0**.**001**
Tricuspid valve variables				
ȃVena contracta, mm	9.4 ± 3.8	9.5 ± 3.5	9.0 ± 4.4	0.210
ȃEROA, mm^2^	51 (29–83)	52 (33–84)	43 (22–75)	0.074
ȃValvular annulus diameter, mm	38.6 ± 7.0	38.2 ± 6.8	39.8 ± 7.4	**0**.**017**
ȃTenting height, mm	8.0 (5.0–12.0)	9.0 (6.0–13.0)	4.9 (2.2–7.0)	**<0**.**001**
ȃTenting area, mm^2^	16 (7–30)	20 (10–34)	8 (4–15)	**<0**.**001**

Values are mean ± SD, median(IQR), or *n*(%). *P*-values < 0.05 were considered statistically significant and are shown in bold.

EROA, effective regurgitant orifice area; FTR, functional tricuspid regurgitation; LV, left ventricle; RA, right atrium; RV, right ventricle; TAPSE, tricuspid annular plane systolic excursion.

When comparing VFTR and AFTR, significant differences were observed in LV ejection fraction, left-sided valvular disease, systolic pulmonary artery pressure, and RV function, inherent to the classification used to define FTR-aetiology. In addition, patients with VFTR showed larger LV and left atrial volumes. Regarding right-sided cardiac variables, as compared with patients with AFTR, patients with VFTR had significantly larger RV dimensions, while showing smaller TV annular diameter (conic RV geometry), and maximal RA dimensions/volumes. Consequently, patients with VFTR presented with more tenting of the TV leaflets (higher tenting height and larger tenting area). No significant differences were observed in TR severity between patients with VFTR and AFTR. The echocardiographic characteristics by the FTR-aetiology subtypes are shown in Supplementary material online, *Table S2*: significant differences were found between VFTR and AFTR, as according to the parameters used to define FTR-aetiology. RV dimensions, RV function, and TV leaflet tenting were also significantly different between AFTR and all VFTR subtypes, while maximal RA dimensions/volumes showed a significant difference only between AFTR and left-sided cardiac disease VFTR.

### Prognostic implications of FTR-aetiologies

**Figure 2:**
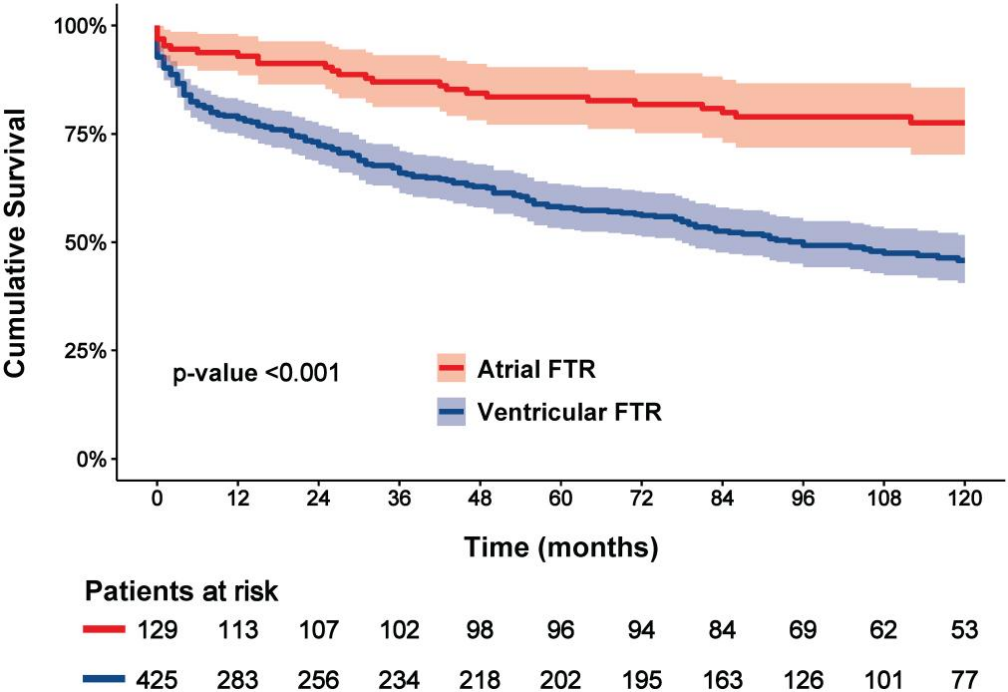
Kaplan–Meier curves for overall survival according to atrial and ventricular FTR. FTR, functional tricuspid regurgitation.

**Figure 3:**
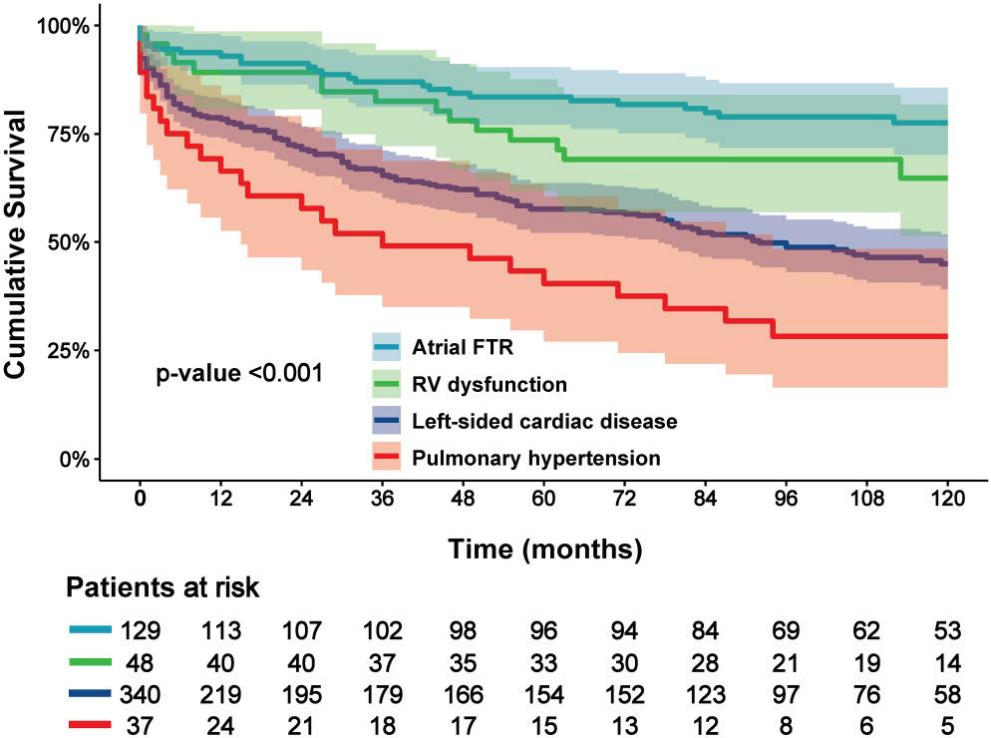
Kaplan–Meier curves for overall survival according to FTR-aetiologies. FTR, functional tricuspid regurgitation.

During a median follow-up of 78 (IQR: 6–116) months, 227 deaths (41%) occurred. The Kaplan–Meier curves for the 10-year overall survival according to VFTR and AFTR as well as according to the four FTR-aetiology subtypes are shown in *Figures 2* and *3*. The cumulative overall survival rates for the total population at 1-, 5-, and 10-year follow-ups were 83, 65, and 54%, respectively. Survival rates at 10-year follow-up were significantly better in patients with AFTR compared with patients with VFTR: 78% vs. 46% (log-rank chi-square: 30.759; *P* < 0.001). Moreover, AFTR had better 10-year overall survival compared with all VFTR-aetiological subtypes: 78, 45, 28, and 65% for AFTR, left-sided cardiac disease, pulmonary hypertension, and RV dysfunction, respectively (log-rank chi-square: 46.163; *P* < 0.001).

Univariable Cox regression analyses for all-cause mortality are presented in Supplementary material online, *Table S3*. On univariable analysis, VFTR was associated with significantly worse outcome compared with AFTR. Multivariable Cox regression analyses for all-cause mortality, following adjustment for the significant clinical and echocardiographic variables on univariable analysis, are presented in *Table 3*. The multivariable analysis showed that patients with VFTR remained independently associated with a worse prognosis compared with patients with AFTR (*Table 3*—model 1) together with older age, male sex, diabetes mellitus, New York Heart Association functional class III or IV, and larger maximal RA volume. The detailed assessment of the FTR-aetiology subtypes showed that all VFTR subtypes were independently associated with worse survival, as compared with AFTR (*Table 3*—model 2). An extended multivariable analysis, also including echocardiographic variables used to define the FTR-aetiologies, showed that patients with VFTR, as well as all VFTR-aetiology subtypes, remained independently associated with worse prognosis, as compared with patients with AFTR (see Supplementary material online, *Table S4*).

**Table 3:** Multivariable Cox proportional hazard models for all-cause mortality in patients with atrial and ventricular FTR

Variable	Model 1		Model 2	
	Hazard ratio (95%CI)	*P*-value	Hazard ratio (95%CI)	*P*-value
Age, years	1.021 (1.007–1.035)	**0**.**003**	1.022 (1.008–1.036)	**0**.**002**
Male sex	1.584 (1.116–2.250)	**0**.**010**	1.586 (1.112–2.264)	**0**.**011**
Diabetes mellitus	1.686 (1.099–2.585)	**0**.**017**	1.643 (1.070–2.522)	**0**.**023**
Dyslipidaemia	0.863 (0.583–1.278)	0.462	0.851 (0.573–1.263)	0.423
Coronary artery disease	1.075 (0.725–1.594)	0.721	1.069 (0.717–1.593)	0.745
NYHA III/IV	1.891 (1.333–2.682)	**<0**.**001**	2.055 (1.423–2.969)	**<0**.**001**
Haemoglobin, g/dL	1.008 (0.928–1.095)	0.847	1.020 (0.937–1.110)	0.651
eGFR-MDRD, mL/min/1.73 m^2^	0.994 (0.988–1.000)	0.064	0.994 (0.988–1.001)	0.091
Loop diuretic	0.949 (0.665–1.355)	0.775	0.945 (0.663–1.347)	0.756
LV end-diastolic volume-indexed, mL/m^2^	1.001 (0.995–1.007)	0.777	1.002 (0.996–1.008)	0.477
Left atrial volume max-indexed, mL/m^2^	0.999 (0.991–1.008)	0.862	0.999 (0.990–1.007)	0.791
RV end-diastolic area-indexed, cm^2^/m^2^	1.012 (0.964–1.063)	0.627	1.003 (0.954–1.055)	0.896
RA volume max-indexed, mL/m^2^	1.012 (1.003–1.022)	**0**.**008**	1.012 (1.003–1.021)	**0**.**010**
EROA, mm^2^	1.002 (0.999–1.005)	0.117	1.002 (1.000–1.005)	0.102
Tenting height, mm	0.992 (0.953–1.033)	0.700	0.985 (0.945–1.026)	0.470
Atrial FTR vs. ventricular FTR	2.929 (1.636–5.244)	**<0**.**001**		
FTR subtypes			—	**<0**.**001**
ȃLeft-sided cardiac disease			2.808 (1.555–5.070)	**0**.**001**
ȃPulmonary hypertension			5.490 (2.544–11.845)	**<0**.**001**
ȃRV dysfunction			2.539 (1.157–5.570)	**0**.**020**
ȃAtrial FTR (reference)			Reference	Reference

Two multivariable Cox models, the first with atrial/ventricular FTR as a dichotomous categorical variable and the second with atrial/ventricular FTR according to the different subgroups. *P*-values < 0.05 were considered statistically significant and are shown in bold.

CI, confidence interval; eGFR-MDRD, estimated glomerular filtration rate—modification of diet in renal disease; EROA, effective regurgitant orifice area; FTR, functional tricuspid regurgitation; LV, left ventricle; NYHA, New York Heart Association functional class; RA, right atrium; RV, right ventricle.

## Discussion

The main findings of the present study can be summarized as follows: (i) From a large series of conservatively treated patients with severe FTR, vast majority is diagnosed with VFTR when applying currently recommended definitions. (ii) By echocardiography, patients with AFTR presented with smaller RV dimensions, larger TV annular diameter, larger maximal RA dimensions/volumes, and less leaflet tenting as compared with patients with VFTR. (iii) AFTR was independently associated with a better long-term prognosis as compared with patients with VFTR, including all VFTR subtypes, after correcting for relevant clinical and echocardiographic variables.

### Clinical and echocardiographic phenotype of FTR-aetiologies

In order to define the aetiology of FTR, some clinical and echocardiographic criteria have been proposed by recent literature,^5^ and the current study applied them in a large cohort with detailed clinical and echocardiographic assessment, in order to further describe their phenotype.

Considering the clinical characteristics, Utsunomiya *et al*. showed in a small cohort that patients diagnosed with AFTR presented with less coronary artery disease, as compared with patients with left-sided cardiac disease; however, without characterization of patients with pulmonary hypertension or RV dysfunction.^11^ This has been confirmed more recently by Gavazzoni *et al*., who additionally showed that patients with AFTR were less symptomatic than VFTR patients.^13^ Schlotter *et al*. expanded to some extent on these results, showing that patients with AFTR (although classified by a different definition) had less heart failure symptoms, less chronic obstructive pulmonary disease, and better kidney function as compared with patients with VFTR.^15^ The results of the current study are in agreement with these findings showing that patients with AFTR also had less cardiovascular comorbidities, higher haemoglobin levels, and less frequent use of loop diuretics, as compared with patients with VFTR.

Considering the morphological characteristics^9–12^ by using two- and three-dimensional echocardiography in a cohort of 113 patients, Florescu *et al*. have shown that patients diagnosed with AFTR present with a conical deformation of the RV due to RV basal enlargement with negligible remodelling of the rest of the RV. Conversely, patients diagnosed with VFTR present with a more elliptic or spherical RV remodelling pattern and a dysfunctional RV due to enlargement of the RV mid diameters and RV lengths.^9^ Moreover, the same research group showed that patients with AFTR had larger TV annulus area than VFTR.^13^ Additionally, significant differences in RA volumes as well as tenting height and tenting area/volume have been identified between AFTR and VFTR.^10–13,15^ The study by Schlotter *et al*.^20^ also showed smaller RV size and less TV tenting area and height, however, with smaller TV annular size and, enlarged, but lower RA area in patients with AFTR as compared with VFTR. The results of the current study are more in line with the study of Florescu and Gavazzoni, and showed that patients with AFTR present with smaller RV dimensions, apart from the basal RV diameter (corresponding to the conical RV shape), better RV function, larger TV annular diameter, larger RA dimensions, and less leaflet tenting as compared with patients with VFTR. As this novel classification takes into account the underlying clinical, anatomical, and pathophysiological differences causing FTR, using parameters easy to obtain and widely available, we believe that it should be systematically used when evaluating FTR-aetiology, with possibly important implications in patient management.

### Survival by FTR-aetiology

Significant TR has been associated with worse outcomes, independently of right-sided heart failure or pulmonary pressures, with a 1- and 5-year overall survival rate of 72.0 and 47.7%, respectively.^2,3,14^ Thus far, survival rates of the newly recommended classification in AFTR and VFTR have not been widely investigated and the prognostic implications of the different FTR-aetiologies remain largely unexplored. Nevertheless, previous studies, seeking to identify prognostic factors in patients diagnosed with significant TR, evaluated some specific variables of underlying heart disease (such as left-sided heart failure, left-sided valvular disease, pulmonary hypertension, or RV dysfunction), which correspond potentially to the respective VFTR subtypes, however without exploring the prognostic difference between AFTR and VFTR.^21–25^ Conversely, studies have investigated isolated TR (i.e. FTR without any other associated left-sided cardiac disease, pulmonary hypertension, or significant comorbidities) in patients with and without AF, yet without comparison to VFTR.^26,27^ For example, evaluating 353 patients with isolated TR, Topilsky *et al*. found a survival rate at a 10-years follow-up of 63 ± 5%, which is comparable to the 10-year survival of the AFTR cohort in the current study.^26^ In a study by Wang *et al*., prognosis of organic and functional TR was compared in a large population, showing that patients with FTR presented greater mortality, although only in an unadjusted analysis.^14^ Of note, patients with FTR had considerably worse survival, as compared with the findings in the current study (30% vs. 54% 10-year overall survival, respectively), highlighting that the less strict selection of VFTR (including, for example, patients with CIED) has a substantial impact on mortality-rate.^14^ Furthermore, Schlotter *et al*. applied a bioinformatic strategy to define AFTR, finally including only three parameters for FTR-classification: TV tenting height, RV midventricular diameter, and LV ejection fraction. They showed that AFTR had a lower rate of the combined endpoint in both the conservative and TTVR cohort as compared with VFTR. Additionally, in analysis limited to the TTVR cohort, VFTR remained independently associated with the composite endpoint at 1-year follow-up, although important discriminators, such as AF and systolic pulmonary artery pressure, were not accounted for in the applied definition.^15^ Noteworthy, both Wang *et al*. and Schlotter *et al*. included patients with VFTR in sinus rhythm as well as AF (leading to possible ambiguity in the true aetiology of VFTR) and, moreover, also included patients with a CIED, despite reflecting patients at higher risk and not correcting for this in the final multivariable model.^14,15^ In both studies, the prognostic value of the different FTR-aetiologies *per se*, as well as several determinative echocardiographic parameters, distinguishing the different FTR-aetiologies, were not specifically assessed in a multivariable Cox analysis when evaluating all-cause death, generating results subject to debate.^14,15^ Additionally, Gavazzoni *et al*. showed in a small cohort, that patients with AFTR had a significantly lower incidence of the combined endpoint (death and heart failure hospitalization) at 1-year follow-up.^13^ The current study expands on this concept by evaluating at long-term follow-up the prognostic implications of the different FTR-aetiologies as such, particularly taking into account important determinative clinical and echocardiographic parameters, and showing that among patients diagnosed with severe FTR, AFTR remains independently associated with better long-term overall survival compared with patients diagnosed with VFTR. Of note, Muraru *et al*. already showed that RA volume is the most important determinant of TV annular diameter with great predictive value for severe FTR.^12^ In addition, the current study shows that RA volume is the only echocardiographic variable, along with the FTR subtypes, that remains independently associated with all-cause mortality.

### Clinical implications

Patients with severe FTR present an absolute increased risk of all-cause mortality, but they are also often referred too late for interventions with an associated high risk of operation, therefore challenging clinicians in their decision making. Identification of parameters which may improve risk stratification in these patients is therefore crucial to achieve both early identification and referral to intervention.^28–32^ Systematic application of the currently recommended classification of FTR-aetiology, differentiating AFTR from VFTR based on specific clinical and echocardiographic parameters, represents both a simple but comprehensive approach which showed to help identify AFTR patients as a relative lower risk group, compared with VFTR patients with increased pulmonary pressures or combined LV dysfunction as the highest risk group. Consequently, this classification could potentially improve patient management in terms of need for close monitoring and potential referral for TV interventions. In patients with AFTR specifically, the relative lower risk, combined with the specific anatomical and functional characteristics, makes them potentially the best candidates for referral to prompt intervention, either surgically or transcatheter. Further research is warranted to define the optimal timing for TV interventions as well as to evaluate the effect and durability of surgical and transcatheter TV repair according to the FTR-aetiology.

### Study limitations

First, this study is subject to limitations of its retrospective observational design from a single tertiary centre and the results need to be confirmed in larger, prospective cohorts. Second, TV and RV parameters were evaluated only with two-dimensional echocardiography, which may underestimate true RV size or TR severity. Three-dimensional echocardiography has shown better correlation with cardiac magnetic resonance for these measurements^33^ and may be recommended for future studies. However, three-dimensional echocardiography was not available in the majority of patients and the proposed approach also seems to be easier to be adopted in the clinical practice for a large-scale screening of these patients. Third, during the course of the long inclusion period the understanding of TR pathophysiology and consequently its treatment has evolved and therefore may have had an impact on outcome. Fourth, some of the VFTR-subgroups had few patients included, limiting the power of analyses involving them. Fifth, granular data were not available to differentiate between cardiac and non-cardiac death. Finally, the classification system of FTR-aetiology was based on the latest recommendations; however, some patients may be wrongly classified when having more than one of the FTR-aetiologies. Therefore, in clinical practice, a patient-tailored approach is advised, yet based on population-based observations.

## Conclusions

Identifying FTR-aetiology, and particularly distinguishing AFTR from VFTR, is of clinical importance, as patients diagnosed with AFTR presented with specific anatomic and pathophysiological characteristics, and most importantly significant better overall survival.

## Supplementary material

Supplementary materials are available at *European Heart Journal – Cardiovascular Imaging* online.

## Supplementary Material

jead016_Supplementary_Data

## Data Availability

The data underlying this article will be shared on reasonable request to the corresponding author.
