# Supplementary material for: Prognostic implications of atrial vs. ventricular functional tricuspid regurgitation
Source: Eur Heart J Cardiovasc Imaging. 2023 Feb 10;24(6):733–41. doi: 10.1093/ehjci/jead016 (PMC10437306; doi:10.1093/ehjci/jead016)
Supplement: jead016_Supplementary_Data [file jead016_supplementary_data.docx]

**SUPLEMENTARY MATERIAL**

**Table S1.** Clinical characteristics of the overall population and according to the FTR aetiologies.

| **Clinical characteristics** | **Overall population**  **n = 554** | **Left-sided cardiac disease**  **n = 340** | **Pulmonary hypertension**  **n = 37** | **RV dysfunction**  **n = 48** | **Atrial FTR**  **n = 129** | **p-value** |
| --- | --- | --- | --- | --- | --- | --- |
| **Demographic characteristics** | | | | | | |
| Age, years | 66 ± 14 | 65 ± 15 | 65 ± 15 | 68 ± 11 | 67 ± 14 | 0.556 |
| Male sex, n (%) | 231 (42) | 152 (45) | 19 (51) | 15 (31) | 45 (35) | 0.063 |
| Body Mass Index, kg/m^2^ | 25.4 ± 4.5 | 25.6 ± 4.7 | 23.8 ± 3.8 | 25.2 ± 4.2 | 25.6 ± 4.0 | 0.140 |
| Systolic blood pressure, mmHg | 127.2 ± 25.3 | 123.4 ± 25.5 | 134.5 ± 30.1 | 131.8 ± 27.3 | 133.4 ± 20.1* | **0.001** |
| Diastolic blood pressure, mmHg | 72.9 ± 15.1 | 71.4 ± 15.5 | 73.7 ± 16.6 | 75.2 ± 11.7 | 75.8 ± 14.2 | 0.063 |
| **Medical history** | | | | | | |
| Arterial hypertension, n (%) | 365 (69) | 229 (71) | 23 (62) | 32 (70) | 81 (63) | 0.317 |
| Diabetes mellitus, n (%) | 96 (18) | 67 (21) | 7 (19) | 9 (20) | 13 (10)* | 0.067 |
| Dyslipidaemia, n (%) | 214 (43) | 149 (48) | 13 (41) | 18 (43) | 34 (30)* | **0.009** |
| Smoking, n (%) | 165 (33) | 109 (35) | 11 (36) | 15 (36) | 30 (26) | 0.348 |
| Coronary artery disease, n (%) | 197 (36) | 140 (42) | 12 (32) | 18 (38) | 27 (21)* | **<0.001** |
| Atrial fibrillation, n (%) | 58 (11) | 0 (0) | 0 (0) | 0 (0) | 58 (45)*†‡ | **<0.001** |
| Chronic kidney disease, n (%) | 89 (17) | 54 (17) | 8 (22) | 9 (20) | 18 (14) | 0.666 |
| COPD, n (%) | 82 (15) | 51 (16) | 4 (11) | 5 (11) | 22 (17) | 0.644 |
| NYHA III or IV, n (%) | 229 (45) | 182 (58) | 13 (38) | 10 (22)* | 24 (20)* | **<0.001** |
| **Laboratory values** | | | | | | |
| Haemoglobin, g/dL | 12.3 ± 2.2 | 12.1 ± 2.2 | 11.8 ± 2.7 | 12.2 ± 2.4 | 12.8 ± 1.9* | **0.015** |
| eGFR MDRD, ml/min/1.73m^2^ | 70.9 ± 31.6 | 68.1 ± 31.7 | 70.6 ± 34.4 | 74.5 ± 35.4 | 77.2 ± 27.9* | 0.051 |
| **Medication** | | | | | | |
| Beta-blocker, n (%) | 253 (51) | 159 (52) | 19 (61) | 22 (54) | 53 (46) | 0.452 |
| RAAS-inh, n (%) | 246 (50) | 164 (54) | 13 (42) | 21 (51) | 48 (42) | 0.127 |
| MRA, n (%) | 84 (17) | 61 (20) | 5 (16) | 2 (5) | 16 (14) | 0.079 |
| Loop diuretic, n (%) | 253 (48) | 183 (56) | 15 (42) | 14 (31)* | 41 (33)* | **<0.001** |

Values are mean ± SD, median (IQR), or n (%). Bonferroni correction: ^*^p<0.05 vs Left-sided cardiac disease; ^†^p<0.05 vs PHTN; ^‡^p<0.05 vs RV dysfunction.

**Abbreviations:** COPD, chronic obstructive pulmonary disease; eGFR MDRD, estimated glomerular filtration rate based on the Modification of Diet in Renal Disease formula; FTR, functional tricuspid regurgitation; IQR, interquartile range; MRA, mineralocorticoid receptor antagonist; NYHA, New York Heart Association functional class; RAAS-inh, Renin-angiotensin-aldosterone system inhibitors; and RV, right ventricle.

**Table S2.** Echocardiographic characteristics of the overall population and according to the FTR aetiologies.

| **Echocardiographic characteristics** | **Overall population**  **n = 554** | **Left-sided cardiac disease**  **n = 340** | **Pulmonary hypertension**  **n = 37** | **RV dysfunction**  **n = 48** | **Atrial FTR**  **n = 129** | **p-value** |
| --- | --- | --- | --- | --- | --- | --- |
| **Left-sided cardiac variables** | | | | | | |
| LV end-diastolic volume - indexed, ml/m^2^ | 56.6 (42.9 – 81.3) | 65.6 (47.0 – 92.8) | 45.8 (32.0 – 74.0)* | 46.4 (38.2 – 58.4)* | 52.3 (38.8 – 64.0)* | **<0.001** |
| LV end-systolic volume – indexed, ml/m^2^ | 27.8 (18.3 – 45.1) | 38.5 (25.7 – 63.1) | 17.0 (12.3 – 28.3)* | 19.1 (14.9 – 24.7)* | 19.7 (15.4 – 26.2)* | **<0.001** |
| LV ejection fraction, % | 48.1 ± 16.0 | 40.3 ± 15.0 | 61.2 ± 8.7* | 59.3 ± 7.1* | 60.6 ± 6.8* | **<0.001** |
| Left-sided valvular disease, n (%) | 199 (36) | 199 (59) | 0 (0)* | 0 (0)* | 0 (0)* | **<0.001** |
| Left atrial volume max - indexed, ml/m^2^ | 39.7 (27.4 – 54.1) | 43.9 (30.5 – 58.1) | 40.4 (26.5 – 64.4) | 31.0 (22.7 – 38.8)* | 36.0 (23.7 – 46.7)* | **<0.001** |
| **Right-sided cardiac variables** | | | | | | |
| RV basal diameter, mm | 44.7 ± 8.6 | 45.3 ± 8.6 | 43.9 ± 9.3 | 42.4 ± 7.9 | 44.1 ± 8.5 | 0.124 |
| RV mid diameter, mm | 34.6 ± 8.9 | 35.0 ± 8.7 | 38.0 ± 9.8 | 34.1 ± 9.8 | 32.9 ± 8.4† | **0.011** |
| RV base-to-apex length, mm | 72.0 ± 11.6 | 73.4 ± 12.0 | 72.3 ± 12.2 | 69.1 ± 10.6 | 69.3 ± 10.1* | **0.002** |
| RV end-diastolic area - indexed, cm^2^/m^2^ | 12.9 ± 4.1 | 13.3 ± 4.1 | 14.7 ± 4.8 | 12.1 ± 4.0† | 11.6 ± 3.4*† | **<0.001** |
| Fractional area change, % | 35.9 ± 13.5 | 32.8 ± 13.3 | 35.7 ± 14.6 | 31.8 ± 11.7 | 45.6 ± 8.9*†‡ | **<0.001** |
| TAPSE, mm | 16.6 ± 5.5 | 15.6 ± 5.2 | 17.1 ± 5.5 | 13.8 ± 4.9† | 20.5 ± 4.6*†‡ | **<0.001** |
| Systolic pulmonary artery pressure, mmHg | 43.0 ± 16.7 | 45.4 ± 16.9 | 65.9 ± 18.2* | 34.9 ± 7.6*† | 33.7 ± 8.6*† | **<0.001** |
| RA area max - indexed, cm^2^/m^2^ | 12.6 ± 4.0 | 12.0 ± 3.7 | 13.2 ± 3.5 | 12.7 ± 3.7 | 14.0 ± 4.5* | **0.001** |
| RA major axis, cm | 5.7 ± 1.0 | 5.6 ± 0.9 | 5.8 ± 1.0 | 5.6 ± 0.8 | 6.0 ± 1.1* | **0.001** |
| RA minor axis, cm | 4.7 ± 1.0 | 4.7 ± 0.9 | 4.7 ± 1.1 | 4.8 ± 1.0 | 5.0 ± 1.1* | **0.007** |
| RA volume max - indexed, ml/m^2^ | 36.8 (27.1 – 52.4) | 35.5 (24.8 – 50.3) | 37.9 (31.8 – 57.6) | 37.0 (27.8 – 50.4) | 41.4 (32.2 – 58.2)* | **0.003** |
| **Tricuspid valve variables** | | | | | | |
| Vena contracta, mm | 9.4 ± 3.8 | 9.4 ± 3.5 | 10.6 ± 3.3 | 9.5 ± 3.8 | 9.0 ± 4.4 | 0.159 |
| EROA, mm^2^ | 51 (29 – 83) | 51 (29 – 83) | 52 (41 – 82) | 60 (35 – 90) | 43 (22 – 75) | 0.188 |
| Valvular annulus diameter, mm | 38.6 ± 7.0 | 38.0 ± 6.7 | 40.2 ± 8.2 | 37.7 ± 6.1 | 39.8 ± 7.4 | **0.028** |
| Tenting height, mm | 8.0 (5.0 – 12.0) | 9.0 (6.0 – 13.0) | 10.6 (6.9 – 15.0) | 9.9 (5.5 – 12.0) | 4.9 (2.2 – 7.0)*†‡ | **<0.001** |
| Tenting area, mm^2^ | 16 (7 – 30) | 19 (10 - 34) | 24 (10 - 43) | 20 (10 - 28) | 8 (4 – 15)*†‡ | **<0.001** |

Values are mean ± SD, median (IQR), or n (%). Bonferroni correction: ^*^p<0.05 vs Left-sided cardiac disease; ^†^p<0.05 vs PHTN; ^‡^p<0.05 vs RV dysfunction.

**Abbreviations:** EROA, effective regurgitant orifice area; LV, left ventricle; PISA, proximal isovelocity surface area; RA, right atrium; RV, right ventricle; and TAPSE, tricuspid annular plane systolic excursion.

**Table S3.** Univariable Cox proportional hazard models for all-cause mortality in patients with atrial and ventricular FTR.

| **Variable** | **Univariable analysis** | |
| --- | --- | --- |
|  | **Hazard Ratio (95%CI)** | **p-value** |
| Age, years | 1.013 (1.003 – 1.023) | **0.009** |
| Male sex | 1.708 (1.315 – 2.217) | **<0.001** |
| Body mass index, kg/m^2^ | 0.976 (0.945 – 1.009) | 0.150 |
| Arterial hypertension | 1.196 (0.890 – 1.607) | 0.236 |
| Diabetes mellitus | 1.786 (1.316 – 2.424) | **<0.001** |
| Dyslipidaemia | 1.457 (1.111 – 1.912) | **0.007** |
| Smoking | 1.280 (0.965 – 1.699) | 0.087 |
| Coronary artery disease | 1.630 (1.252 – 2.122) | **<0.001** |
| Chronic kidney disease | 2.177 (1.591 – 2.978) | **<0.001** |
| COPD | 1.399 (0.990 – 1.976) | 0.057 |
| NYHA III or IV | 2.537 (1.931 – 3.335) | **<0.001** |
| Haemoglobin, g/dL | 0.924 (0.869 – 0.981) | **0.010** |
| eGFR MDRD, ml/min/1.73m^2^ | 0.988 (0.984 – 0.993) | **<0.001** |
| Previous cardiac surgery | 1.084 (0.831 – 1.415) | 0.551 |
| Loop diuretic | 1.561 (1.197 – 2.036) | **0.001** |
| LV end-diastolic volume - indexed, ml/m^2^ | 1.007 (1.003 – 1.011) | **0.001** |
| LV end-systolic volume - indexed, ml/m^2^ | 1.010 (1.005 – 1.015) | **<0.001** |
| LV ejection fraction, % | 0.986 (0.978 – 0.994) | **0.001** |
| Left-sided valvular disease | 1.612 (1.234 – 2.105) | **<0.001** |
| Left atrial volume max - indexed, ml/m^2^ | 1.008 (1.003 – 1.014) | **0.004** |
| RV basal diameter, mm | 1.021 (1.006 – 1.036) | **0.006** |
| RV mid diameter, mm | 1.027 (1.012 – 1.042) | **<0.001** |
| RV length, mm | 1.019 (1.008 – 1.031) | **0.001** |
| RV end-diastolic area - indexed, cm^2^/m^2^ | 1.099 (1.067 – 1.133) | **<0.001** |
| RV fractional area change, % | 0.980 (0.971 – 0.989) | **<0.001** |
| TAPSE, mm | 0.956 (0.932 – 0.981) | **0.001** |
| Systolic pulmonary artery pressure, mmHg | 1.026 (1.018 – 1.034) | **<0.001** |
| RA area max - indexed, cm^2^/m^2^ | 1.035 (1.005 – 1.067) | **0.024** |
| RA volume max - indexed, ml/m^2^ | 1.008 (1.003 – 1.013) | **0.002** |
| EROA, mm^2^ | 1.003 (1.001 – 1.005) | **0.001** |
| Valvular annulus diameter, mm | 1.019 (1.000 – 1.037) | **0.046** |
| Tenting height, mm | 1.050 (1.025 – 1.075) | **<0.001** |
| Tenting area, mm^2^ | 1.187 (1.111 – 1.267) | **<0.001** |
| Atrial FTR vs ventricular FTR | 2.922 (1.977 – 4.321) | **<0.001** |
| FTR subtypes | - | **<0.001** |
| Left-sided cardiac disease | 2.979 (2.001 – 4.437) | **<0.001** |
| Pulmonary hypertension | 4.945 (2.906 – 8.414) | **<0.001** |
| RV dysfunction | 1.667 (0.916 – 3.033) | 0.095 |
| Atrial FTR (reference) | Ref. | Ref. |

**Abbreviations:** CI, confidence interval; COPD, chronic obstructive pulmonary disease; eGFR MDRD, estimated glomerular filtration rate based on the Modification of Diet in Renal Disease formula; EROA, effective regurgitant orifice area; FTR, functional tricuspid regurgitation; LV, left ventricle; NYHA, New York Heart Association functional class; RA, right atrium; Ref., reference; RV, right ventricle; and TAPSE, tricuspid annular plane systolic excursion.

**Table S4.** Multivariable Cox proportional hazard models for all-cause mortality in patients with ventricular and atrial functional tricuspid regurgitation.

| **Variable** | **Model 1** | | **Model 2** | |
| --- | --- | --- | --- | --- |
|  | **Hazard Ratio (95%CI)** | **p-value** | **Hazard Ratio (95%CI)** | **p-value** |
| Age, years | 1.021 (1.006 – 1.035) | **0.004** | 1.021 (1.007 – 1.036) | **0.003** |
| Male sex | 1.480 (1.023 – 2.142) | **0.037** | 1.477 (1.013 – 2.154) | **0.043** |
| Diabetes mellitus | 1.503 (0.967 – 2.336) | 0.070 | 1.498 (0.963 – 2.330) | 0.073 |
| Dyslipidaemia | 0.746 (0.497 – 1.121) | 0.159 | 0.742 (0.492 – 1.117) | 0.153 |
| Coronary artery disease | 1.219 (0.809 – 1.838) | 0.344 | 1.165 (0.768 – 1.769) | 0.472 |
| NYHA III or IV | 1.981 (1.365 – 2.875) | **<0.001** | 2.092 (1.432 – 3.056) | **<0.001** |
| Haemoglobin, g/dL | 1.026 (0.942 – 1.117) | 0.557 | 1.040 (0.953 – 1.135) | 0.377 |
| eGFR MDRD, ml/min/1.73m^2^ | 0.994 (0.988 – 1.001) | 0.082 | 0.994 (0.988 – 1.001) | 0.103 |
| Loop diuretic | 0.935 (0.645 – 1.355) | 0.722 | 0.915 (0.632 – 1.326) | 0.639 |
| LV end-diastolic volume - indexed, ml/m^2^ | 1.001 (0.995 – 1.008) | 0.731 | 1.001 (0.995 – 1.008) | 0.687 |
| LV ejection fraction, % | 1.008 (0.993 – 1.023) | 0.278 | 1.002 (0.984 – 1.019) | 0.863 |
| Left-sided valvular disease | 0.944 (0.645 – 1.383) | 0.768 | 1.090 (0.704 – 1.688) | 0.699 |
| Left atrial volume max - indexed, ml/m^2^ | 0.995 (0.986 – 1.004) | 0.277 | 0.995 (0.986 – 1.004) | 0.249 |
| RV end-diastolic area - indexed, cm^2^/m^2^ | 1.006 (0.957 – 1.058) | 0.811 | 0.999 (0.949 – 1.050) | 0.954 |
| RV fractional area change, % | 0.990 (0.976 – 1.005) | 0.183 | 0.989 (0.975 – 1.003) | 0.135 |
| RA volume max - indexed, ml/m^2^ | 1.017 (1.006 – 1.028) | **0.001** | 1.017 (1.007 – 1.026) | **<0.001** |
| Systolic pulmonary artery pressure, mmHg | 1.017 (1.006 – 1.028) | **0.002** | 1.016 (1.004 – 1.027) | **0.007** |
| EROA, mm^2^ | 1.003 (1.000 – 1.006) | **0.047** | 1.003 (1.000 – 1.006) | **0.022** |
| Tenting height, mm | 0.991 (0.952 – 1.031) | 0.650 | 0.984 (0.944 – 1.025) | 0.429 |
| Atrial FTR vs ventricular FTR | 2.650 (1.369 – 5.129) | **0.004** |  |  |
| FTR subtypes |  |  | - | **0.003** |
| Left-sided cardiac disease |  |  | 2.223 (1.042 – 4.744) | **0.039** |
| Pulmonary hypertension |  |  | 4.795 (2.093 – 10.987) | **<0.001** |
| RV dysfunction |  |  | 2.282 (1.012 – 5.148) | **0.047** |
| Atrial FTR (reference) |  |  | Ref. | Ref. |

Two multivariable Cox models, the first with atrial/ventricular FTR as a dichotomous categorical variable and the second with atrial/ventricular FTR according to the different subgroups.

**Abbreviations:** CI, confidence interval; eGFR MDRD, estimated glomerular filtration rate based on the Modification of Diet in Renal Disease formula; EROA, effective regurgitant orifice area; FTR, functional tricuspid regurgitation; LV, left ventricle; NYHA, New York Heart Association functional class; RA, right atrium; Ref., reference; and RV, right ventricle.
